# A *PEX5* missense allele preferentially disrupts PTS1 cargo import into Arabidopsis peroxisomes

**DOI:** 10.1002/pld3.128

**Published:** 2019-03-20

**Authors:** Khushali J. Patel, Yun‐Ting Kao, Roxanna J. Llinas, Bonnie Bartel

**Affiliations:** ^1^ Department of BioSciences Rice University Houston Texas; ^2^ Present address: Department of Cell Biology and Molecular Genetics University of Maryland College Park Maryland; ^3^ Present address: Graduate School of Biomedical Sciences Baylor College of Medicine Houston Texas

**Keywords:** *Arabidopsis thaliana*, peroxin, peroxisome import, peroxisome‐targeting signal, PEX5

## Abstract

The sorting of eukaryotic proteins to various organellar destinations requires receptors that recognize cargo protein targeting signals and facilitate transport into the organelle. One such receptor is the peroxin PEX5, which recruits cytosolic cargo carrying a peroxisome‐targeting signal (PTS) type 1 (PTS1) for delivery into the peroxisomal lumen (matrix). In plants and mammals, PEX5 is also indirectly required for peroxisomal import of proteins carrying a PTS2 signal because PEX5 binds the PTS2 receptor, bringing the associated PTS2 cargo to the peroxisome along with PTS1 cargo. Despite PEX5 being the PTS1 cargo receptor, previously identified Arabidopsis *pex5* mutants display either impairment of both PTS1 and PTS2 import or defects only in PTS2 import. Here, we report the first Arabidopsis *pex5* mutant with an exclusive PTS1 import defect. In addition to markedly diminished GFP‐PTS1 import and decreased pex5‐2 protein accumulation, this *pex5‐2* mutant shows typical peroxisome‐related defects, including inefficient β‐oxidation and reduced growth. Growth at reduced or elevated temperatures ameliorated or exacerbated *pex5‐2* peroxisome‐related defects, respectively, without markedly changing pex5‐2 protein levels. In contrast to the diminished PTS1 import, PTS2 processing was only slightly impaired and PTS2‐GFP import appeared normal in *pex5‐2*. This finding suggests that even minor peroxisomal localization of the PTS1 protein DEG15, the PTS2‐processing protease, is sufficient to maintain robust PTS2 processing.

## 1 INTRODUCTION

Peroxisomes are organelles that are essential for many processes including metabolism, development, and environmental responses (reviewed in Reumann & Bartel, 2016; Kao, Gonzalez, & Bartel, 2018). Peroxisomes are crucial to plant growth as they house fatty acid β‐oxidation (reviewed in Graham, 2008), which provides fixed carbon to seedlings prior to the establishment of photosynthesis. Moreover, several steps in photorespiration, which allows recycling of metabolites when RuBisCO uses oxygen instead of carbon dioxide (reviewed in Dellero, Jossier, Schmitz, Maurino, & Hodges, 2016), are peroxisomal. Peroxisomes can arise either de novo from the endoplasmic reticulum (ER) or via fission of existing peroxisomes (reviewed in Kao et al., 2018). Pre‐peroxisomes derived from the ER (and mitochondria in mammals) can deliver phospholipids and peroxisome membrane proteins (PMPs) to preexisting peroxisomes or fuse to give rise to new peroxisomes (Mullen & Trelease, 2006; Sugiura, Mattie, Prudent, & McBride, 2017; van der Zand, Gent, Braakman, & Tabak, 2012).

Peroxins (PEX proteins) facilitate peroxisome biogenesis, division, and matrix protein import. Mature peroxisomes can posttranslationally import fully folded matrix proteins from the cytosol (Lee, Mullen, & Trelease, 1997; McNew & Goodman, 1994). PEX5 and PEX7 are receptors that recognize matrix proteins carrying either a C‐terminal peroxisome targeting signal (PTS) type 1 (PTS1) or an N‐terminal PTS2, respectively (reviewed in Kao et al., 2018). In yeast, PEX5 interacts with PEX14 at the peroxisome surface to induce transient pores in the membrane that allow cargo import into the matrix (Meinecke et al., 2010). In the peroxisome, the PTS2 signal is cleaved from the cargo by the peroxisomal protease DEG15 in plants (Helm et al., 2007; Schuhmann, Huesgen, Gietl, & Adamska, 2008) or Tysnd1 in mammals (Kurochkin et al., 2007). As DEG15 and Tysnd1 are PTS1 proteins, PTS2 processing presumably requires both PTS1 and PTS2 import. After cargo delivery in yeast, PEX5 in the peroxisomal membrane is ubiquitinated and targeted for recycling or degradation by the peroxisome‐tethered ubiquitin‐conjugating enzyme (Ubc) PEX4 and the RING–finger ubiquitin–protein ligase complex consisting of PEX2, PEX10, and PEX12 (reviewed in Platta, Hagen, Reidick, & Erdmann, 2014). In yeast, PEX5 that is monoubiquitinated by PEX12 and PEX4 is recycled from the peroxisomal membrane by the peroxisome‐tethered PEX1–PEX6 ATPase complex for additional import rounds (Pedrosa et al., 2018; Platta, Grunau, Rosenkranz, Girzalsky, & Erdmann, 2005). In contrast, PEX5 that is polyubiquitinated by PEX2 and the cytosolic Ubc4 is targeted for proteasomal degradation (Platta et al., 2009).

Core peroxins that facilitate matrix protein import are conserved in the reference plant *Arabidopsis thaliana* (reviewed in Kao et al., 2018; Woodward & Bartel, 2018). With the exception of *PEX14* (Hayashi et al., 2000; Monroe‐Augustus et al., 2011), known null alleles of genes encoding peroxins confer embryonic lethality in Arabidopsis (Boisson‐Dernier, Frietsch, Kim, Dizon, & Schroeder, 2008; Fan et al., 2005; Goto, Mano, Nakamori, & Nishimura, 2011; Hu et al., 2002; McDonnell et al., 2016; Schumann, Wanner, Veenhuis, Schmid, & Gietl, 2003; Sparkes et al., 2003). Thus, the roles of most plant peroxins have been elucidated by analyzing partial loss‐of‐function missense alleles (Burkhart, Kao, & Bartel, 2014; Burkhart, Lingard, & Bartel, 2013; Gonzalez et al., 2017; Goto et al., 2011; Kao, Fleming, Ventura, & Bartel, 2016; Mano, Nakamori, Nito, Kondo, & Nishimura, 2006; Ramón & Bartel, 2010; Rinaldi et al., 2017; Woodward et al., 2014; Zolman & Bartel, 2004; Zolman, Monroe‐Augustus, Silva, & Bartel, 2005; Zolman, Yoder, & Bartel, 2000), T‐DNA insertions that incompletely abolish function (Khan & Zolman, 2010; Ratzel, Lingard, Woodward, & Bartel, 2011; Woodward & Bartel, 2005a; Zolman et al., 2005), or RNAi approaches (Fan et al., 2005; Hayashi, Yagi, Nito, Kamada, & Nishimura, 2005; Nito, Kamigaki, Kondo, Hayashi, & Nishimura, 2007; Orth et al., 2007).

**Figure 1:**
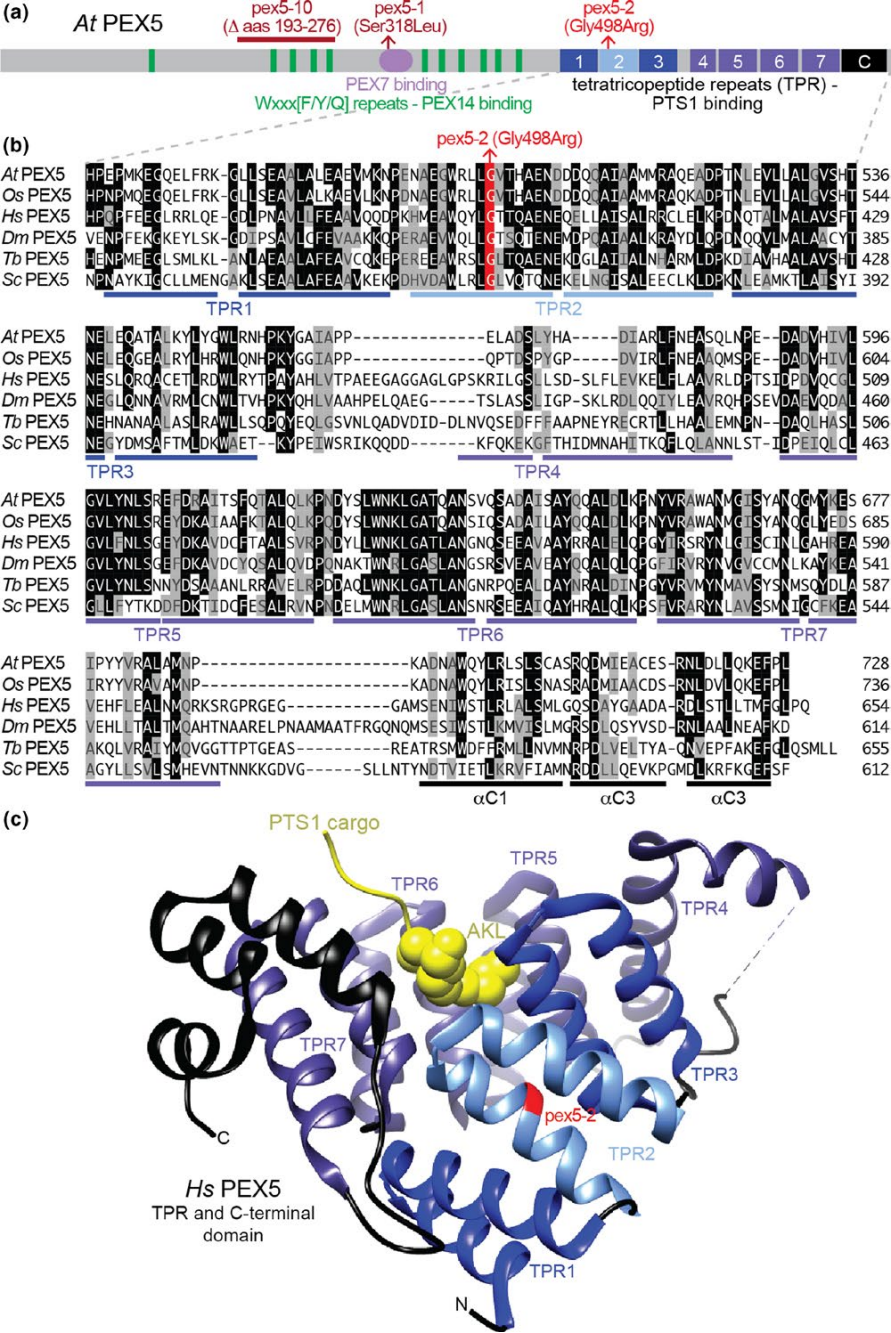
Arabidopsis *pex5* alleles alter different protein domains. (a) Schematic of Arabidopsis (*At*) PEX5 protein showing Wxxx [F/Y/Q] repeats (green) that may bind PEX14, the region corresponding to the PEX7‐binding domain (lavender) in human PEX5 (Dodt, Warren, Becker, Rehling, & Gould, 2001), PTS1‐binding tetratricopeptide repeats (TPR; numbered), the C‐terminal domain (black), and Arabidopsis *pex5* mutations (red). (b) Alignment of the TPR and C‐terminal domains of PEX5 orthologs from *Arabidopsis thaliana* (*At*; OAO90794.1), *Oryza sativ*a (Os; AAX11255.1), *Homo sapiens* (*Hs*; NP_001124495.1), *Drosophila melanogaster* (*Dm*; AAF45676.2), *Trypanosoma brucei* (*Tb*; AAD54220.1), and *Saccharomyces cerevisiae* (*Sc*; KZV12481.1) generated using the Clustal W method of the Megalign program (DNAStar). Identical residues in at least four sequences are highlighted in black, chemically similar residues are in gray, and the pex5‐2 mutation is in red. α‐helices in the human PEX5 structure (panel c) are indicated by bars under the alignment. (c) Structure of the human (*Hs*) PEX5 TPR domain (sequence in panel b) in a complex with a PTS1 cargo protein (yellow). The Gly residue analogous to the residue altered in pex5‐2 is highlighted in red. The ribbon diagram was generated from the PEX5‐sterol carrier protein (SCP2) structure deposited in the Protein Data Bank under ID code 2C0L (Stanley et al., 2006) using UCSF Chimera software (Pettersen et al., 2004)

Analysis of mutants defective in peroxisome cargo receptors can provide insight into the import machinery. Only two Arabidopsis *pex5* mutants, *pex5‐10* and *pex5‐1*, have been described. *pex5‐10* carries a T‐DNA insertion in the fifth exon of *PEX5* (Zolman et al., 2005) that results in the skipping of this exon and production of an internally deleted pex5‐10 protein lacking several predicted PEX14‐binding motifs (Figure 1a) (Khan & Zolman, 2010). The *pex5‐10* mutant, like *pex5* RNAi lines (Hayashi et al., 2005), has defects in both PTS1 and PTS2 import (Khan & Zolman, 2010; Lingard & Bartel, 2009). *pex5‐1* is a missense allele that creates a Ser318Leu substitution (Zolman et al., 2000) in the predicted PEX7‐binding domain (Figure 1a), and the *pex5‐1* mutant has impaired PTS2 import but wild‐type PTS1 import (Woodward & Bartel, 2005a). Similarly, Arabidopsis *pex7* mutants and RNAi lines display defects in PTS2 import (Hayashi et al., 2005; Ramón & Bartel, 2010; Woodward & Bartel, 2005a). In addition to PTS2 import defects, Arabidopsis *pex7* mutants show decreased PEX5 levels and defects in PTS1 import (Ramón & Bartel, 2010), indicating that PEX5 and PEX7 are interdependent. As Arabidopsis *pex5* mutants with exclusively PTS1 import defects have not been reported, distinguishing the functions of PTS1 and PTS2 import in plants has been challenging.

In this study, we describe a novel *pex5* missense mutation (*pex5‐2*) recovered from a forward genetic screen for β‐oxidation defects. The *pex5‐2* mutant exhibited reduced growth, low PEX5 levels, and decreased peroxisomal import of GFP‐PTS1 protein. In contrast, *pex5‐2* displayed robust PTS2‐GFP import and only slight defects in PTS2 protein processing, suggesting that relatively little PTS1 import may be sufficient to efficiently cleave PTS2 signals. Some *pex5‐2* deficiencies were exacerbated at elevated growth temperature and ameliorated at lowered growth temperature, suggesting that PEX5 function and/or pex5‐2 dysfunction is impacted by temperature. The distinct and overlapping defects of the Arabidopsis *pex5‐1*,* pex5‐2*, and *pex5‐10* mutants will allow continued elucidation of the relationships between PTS1 and PTS2 import in plants.

## 2 MATERIALS AND METHODS

### 2.1 Plant materials and growth conditions

Arabidopsis (*Arabidopsis thaliana*) wild type and mutants were in the Columbia‐0 (Col‐0) background. *pex4‐1* (Zolman et al., 2005), *pex5‐1* (Zolman et al., 2000), *pex5‐10* (Zolman et al., 2005), and *pex6‐1* (Zolman & Bartel, 2004) were previously described. Wild type transformed with *35S:PEX5* (Zolman & Bartel, 2004), *35S:GFP‐PTS1* (Zolman & Bartel, 2004), or *35S:PTS2‐GFP* (Woodward & Bartel, 2005a); *pex4‐1* carrying *35S:GFP‐PTS1* (Zolman et al., 2005); and *pex5‐1* carrying *35S:GFP‐PTS1* (Woodward & Bartel, 2005a) were previously described. *pex5‐1* carrying *35S:PTS2‐GFP*,* pex5‐2* carrying *35S:PTS2‐GFP*, and *pex5‐2* carrying *35S:PEX5* and *35S:GFP‐PTS1* were selected from progeny of the corresponding crosses using PCR‐based genotyping with the primers listed in Supporting Information Table S1. All assays except the initial characterization (Supporting Information Figure S1) used *pex5‐2* carrying *35S:GFP‐PTS1* that had been backcrossed at least once with wild type carrying *35S:GFP‐PTS1*.

Seeds were surface‐sterilized with bleach solution (3% NaOCl and 0.01% Triton X‐100), washed twice with sterile water, suspended in 0.1% (w/v) agar, and stratified in dark at 4°C for 1–3 days. Stratified seeds were plated on plant nutrient (PN) media (Haughn & Somerville, 1986) solidified with 0.6% (w/v) agar and supplemented with 0.5% sucrose (PNS) as indicated. PNS plates were supplemented with IBA (from a 100 mM IBA stock in ethanol) as indicated. Plates were incubated at 22°C in yellow‐filtered light (Stasinopoulos & Hangarter, 1990) for the indicated number of days, and light‐grown root lengths were measured and/or seedlings were used for immunoblotting or confocal microscopy. For temperature experiments, plates were incubated at 22°C in yellow‐filtered light for 1 day and then incubated at 16, 22, or 28°C for seven additional days for light‐grown experiments or wrapped in aluminum foil and grown at 16, 22, or 28°C for four additional days for dark‐grown experiments. All experiments except the initial characterization (Supporting Information Figure S1) were repeated at least twice with similar results; representative results are shown.

### 2.2 *pex5‐2* isolation

Ethyl methanesulfonate (EMS) mutagenesis of wild‐type seeds carrying *35S:GFP‐PTS1* was previously described (Rinaldi et al., 2016). M_2_ seeds were grown for about 2 weeks in yellow‐filtered light on PNS supplemented with 100 mM NaCl and 12 μM IBA, and putative mutants with elongated roots were transferred to soil for seed production. M_3_ lines displaying resistance to 10 μM IBA (with or without 100 mM NaCl) and wild‐type‐like sensitivity to 80 nM 2,4‐dichlorophenoxyacetic acid were retained for further analysis.

### 2.3 Whole‐genome sequencing

Approximately 100 *pex5‐2* M_3_ seeds were plated on PNS covered with sterile filter paper. Genomic DNA was purified (Thole, Beisner, Liu, Venkova, & Strader, 2014) from 18‐day‐old light‐grown seedlings and sequenced by the Genome Technology Access Center at Washington University in St. Louis. Sequence reads were aligned with the TAIR 10 build of the *A. thaliana* Col‐0 genome using Novoalign (Novocraft; http://novocraft.com). SNPs were identified using SAMtools (Li, 2011; Li et al., 2009) and annotated with snpEFF (Cingolani et al., 2012). Mutations were filtered using a script prioritizing homozygous EMS‐derived mutations (G‐to‐A and C‐to‐T) causing non‐synonymous amino acid changes or altering splice sites. We disregarded mutations that were present in our laboratory stock of wild‐type Col‐0. Identifiers of genes with homozygous EMS‐consistent mutations are displayed in Supporting Information Figure S2.

### 2.4 Immunoblot analysis

Seedling extracts were processed for immunoblotting as described (Kao & Bartel, 2015). Membranes were incubated overnight with rabbit primary antibodies raised against PMDH2 (1:5,000; Pracharoenwattana, Cornah, & Smith, 2007), the PED1 isoform of thiolase (1:10,000; Lingard, Monroe‐Augustus, & Bartel, 2009), or PEX5 (1:100; Zolman & Bartel, 2004) followed by horseradish peroxidase (HRP)‐linked goat anti‐rabbit secondary antibody (1:5,000 dilution of 0.125 mg/ml, GenScript A00098) or with mouse primary antibodies raised against HSC70 (1:100,000, Stressgen HPA‐817) followed by HRP‐linked goat anti‐mouse secondary antibody (1:5,000, Santa Cruz Biotechnology sc‐2031). Antibodies were visualized using enhanced chemiluminescent HRP substrate (Advansta Western Bright K‐12045 or Prometheus ProSignal Pico) and exposed on autoradiography film. Membranes were sequentially probed with various antibodies without stripping. Representative films were scanned with a flatbed scanner, and band intensity was quantified using ImageJ (version 1.42q; Schneider, Rasband, & Eliceiri, 2012).

### 2.5 Confocal fluorescence microscopy

Light‐grown seedlings carrying *35S:GFP‐PTS1* or *35S:PTS2‐GFP* were mounted in water and imaged using a Carl Zeiss LSM 710 laser scanning confocal microscope equipped with a meta detector and 40× Plan‐Apochromat oil‐immersion objectives. GFP was excited with a 488‐nm argon laser and fluorescence was collected between 493 and 552 nm. Each image corresponds to a 1.0‐μm optical section (pinhole = 1 airy unit) and is an average of four exposures.

For quantification of confocal images (collected at a 1,024 × 1,024 pixel setting; 4.8177 pixels/μm), ImageJ macros (listed in Supporting Information) were used to measure the intensity of punctate fluorescence [defined as particles of at least 15 pixels (3.11 μm) in diameter with a circularity of 0.2–1.0] and the total intensity in the image [defined as particles of at least 2 pixels (0.415 μm) in diameter with circularity of 0–1.0]. The fraction of [total punctate intensity]/[total intensity] from three images (Supporting Information Figures S3 and S4) was calculated as a measure of peroxisomal import.

### 2.6 Statistical analysis

SPSS Statistics software (Version 24.0.0.0) was used to analyze measurements. One‐way analysis of variance (ANOVA) followed by the Duncan's post hoc test was used to determine the significance of differences among genotypes or treatments.

## 3 RESULTS

### 3.1 *pex5‐2* displays peroxisome‐related defects that are rescued by PEX5 overexpression

As β‐oxidation is exclusively peroxisomal in Arabidopsis (reviewed in Graham, 2008), we can use β‐oxidation efficiency to assess peroxisome function (reviewed in Bartel, Burkhart, & Fleming, 2014). The predominant naturally occurring auxin, indole‐3‐acetic acid (IAA), plays critical roles in plant growth and development by modulating cell identity, division, and elongation (Woodward & Bartel, 2005b). One IAA precursor, indole‐3‐butyric acid (IBA), is converted into IAA via β‐oxidation in peroxisomes (Strader & Bartel, 2011; Strader, Culler, Cohen, & Bartel, 2010). Thus, in seedlings with functioning peroxisomes, IBA treatment confers high‐auxin phenotypes including reduced root and hypocotyl elongation (Strader et al., 2011; Zolman et al., 2000), and IBA‐resistance screens have uncovered genes needed for peroxisome biogenesis and function (reviewed in Bartel et al., 2014). As salt increases Arabidopsis peroxisome abundance (Fahy et al., 2017; Frick & Strader, 2018; Mitsuya et al., 2010), we reasoned that screening for IBA resistance in the presence of salt might uncover factors impacting salinity‐induced peroxisome proliferation. We therefore identified seedlings with elongated roots on normally inhibitory concentrations of IBA and NaCl. Subsequent analyses of a mutant emerging from this screen showed reduced response to IBA in the presence of NaCl, but this mutant was even less IBA responsive in the absence of NaCl (Supporting Information Figure S1a), suggesting that we had not disrupted a proliferation‐related gene. Moreover, root growth of this mutant was also resistant to inhibition by 2,4‐dichlorophenoxybutryic acid (Supporting Information Figure S1b), which, like IBA, requires peroxisomal chain shortening for activity (Hayashi, Toriyama, Kondo, & Nishimura, 1998). In contrast, the mutant root growth was inhibited similar to wild type by the synthetic auxin 2,4‐dichlorophenoxyacetic acid (Supporting Information Figure S1b), suggesting that general auxin responsiveness was intact. We concluded that the mutant defects stemmed from decreased peroxisome function and we selected the mutant for in‐depth analysis.

Whole‐genome sequencing of genomic DNA from this mutant revealed a homozygous G‐to‐A missense mutation in the *PEX5* gene (Supporting Information Figure S2), and we named the mutant *pex5‐2* (Figure 1). The *pex5‐2* mutation results in a Gly498Arg substitution in one of the seven tetratricopeptide repeat (TPR) domains (Figure 1) that comprise the PTS1‐binding region of PEX5 (Gatto, Geisbrecht, Gould, & Berg, 2000; Terlecky, Nuttley, McCollum, Sock, & Subramani, 1995). The crystal structure of the human PEX5 TPR domain (Stanley et al., 2006) reveals that each TPR consists of two α‐helices that pack together to form a PTS1‐binding pocket (Figure 1c). The Gly498Arg substitution in *pex5‐2* is in the middle of the first predicted α‐helix of TPR2 (Figure 1b,c) and alters a Gly residue that is conserved in diverse PEX5 orthologs (Figure 1b).

**Figure 2:**
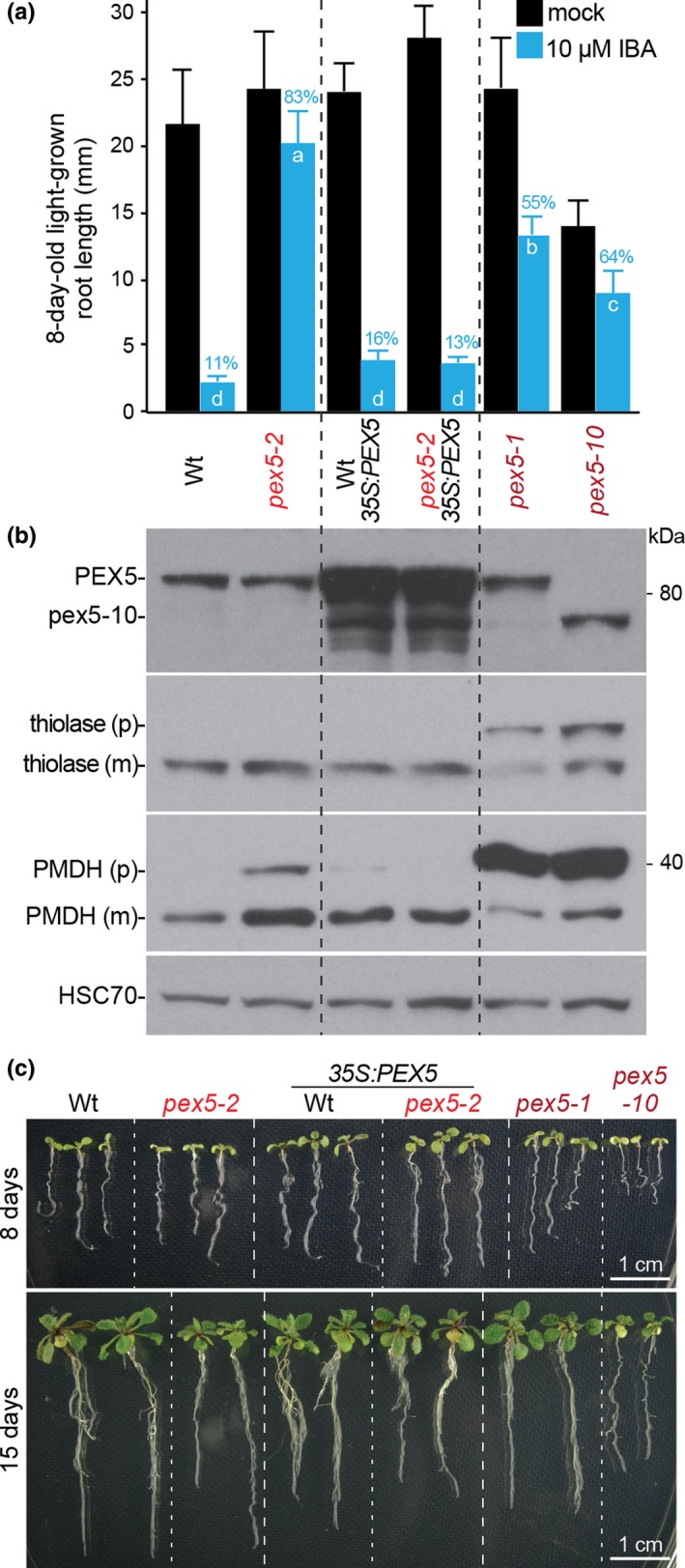
*pex5‐2* displays peroxisome‐related defects that are rescued by *PEX5* overexpression. (a) *pex5‐2* is resistant to the inhibitory effects of IBA on root elongation. Bars indicate mean root lengths of 8‐day‐old seedlings grown in light in the absence or presence of 10 μM IBA on media supplemented with 0.5% sucrose (*n *=* *12). Error bars indicate standard deviations. Different letters within bars indicate significant differences between genotypes following IBA treatment (one‐way ANOVA,* p *<* *0.001). Percentages above bars indicate relative elongation for each line on IBA compared to mock conditions. (b) *pex5‐2* displays a slight defect in PTS2 processing. Extracts from 8‐day‐old light‐grown seedlings were processed for immunoblotting and probed with antibodies to the indicated proteins. PMDH and thiolase are synthesized as precursor (p) proteins that are processed into mature forms (m) after peroxisome entry. HSC70 was used to monitor loading. (c) *pex5‐2* displays slight growth defects. Plants were grown on agar‐based medium containing 0.5% sucrose for 8 or 15 days, and seedlings were moved to a new plate and photographed

In addition to strong IBA resistance (Figure 2a), the *pex5‐2* mutant exhibited a slight defect in processing of the peroxisomal malate dehydrogenase (PMDH) PTS2 protein (Figure 2b). IBA resistance was closely linked to the *pex5‐2* mutation; 12 of 12 IBA‐resistant F_2_ seedlings from a backcross to wild type were *pex5‐2* homozygotes. Moreover, overexpressing wild‐type *PEX5* using the constitutive cauliflower mosaic virus *35S* promoter restored both IBA responsiveness and PTS2 processing to *pex5‐2* seedlings (Figure 2a,b). These linkage and complementation experiments confirmed that the identified *pex5‐2* mutation was causing the observed peroxisome‐related impairments.

We compared *pex5‐2* with previously reported *pex5* alleles: *pex5‐10* and *pex5‐1*. The *pex5‐10* T‐DNA insertion results in an internal deletion removing several of the predicted PEX14‐binding motifs (Khan & Zolman, 2010; Zolman et al., 2005), and *pex5‐1* carries a Ser318Leu substitution in the putative PEX7‐binding domain (Woodward & Bartel, 2005a; Zolman et al., 2000). Like *pex5‐1*, the *pex5‐2* mutant accumulated apparently full‐length PEX5 protein (Figure 2b). Of the three alleles, *pex5‐2* displayed the most robust IBA resistance (Figure 2a) whereas *pex5‐1* and *pex5‐10* displayed more severe PMDH and thiolase PTS2‐processing defects than *pex5‐2* (Figure 2b).

*pex5‐2* also displayed growth defects. Unlike *pex5‐1* shoots, which resembled wild type, *pex5‐2* shoots were smaller than wild type and more similar to *pex5‐10* (Figure 2c). In contrast, *pex5‐2* seedling roots elongated similar to wild type and *pex5‐1* on sucrose‐supplemented media (Figure 2a,c). As with IBA responsiveness (Figure 2a), expressing wild‐type *PEX5* in the *pex5‐2* mutant improved rosette size in the mutant (Figure 2c), indicating that these defects were caused by decreased PEX5 function.

### 3.2 *pex5‐2* exhibits PTS1 but not PTS2 import defects

**Figure 3:**
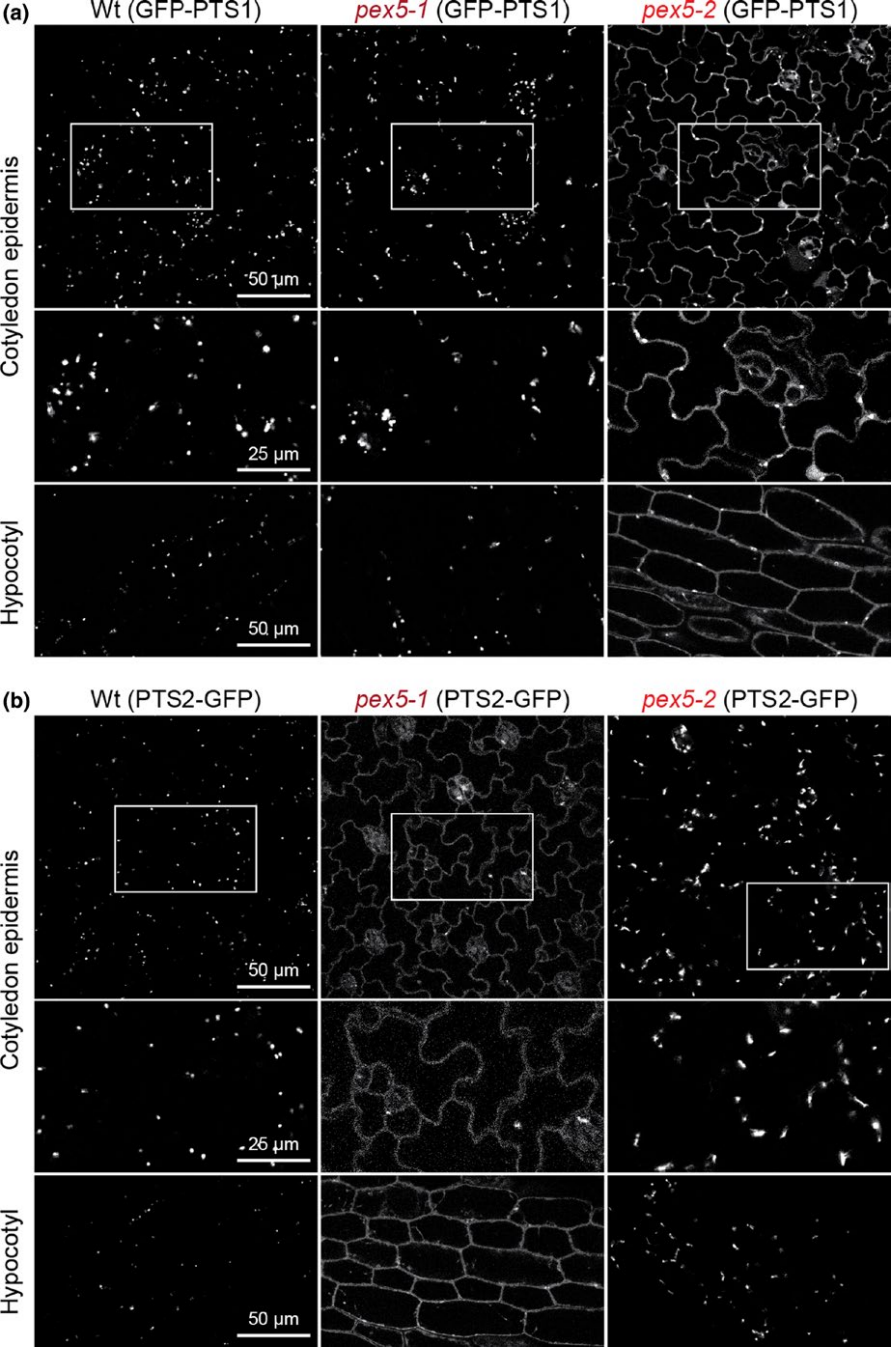
*pex5‐2* displays defective GFP‐PTS1 import (a) and efficient PTS2‐GFP import (b). Cotyledon epidermal cells (top rows) and hypocotyl cells (bottom rows) of 4‐day‐old light‐grown seedlings carrying the *35S:GFP‐PTS1* (Zolman & Bartel, 2004) or *35S:PTS2‐GFP* (Woodward & Bartel, 2005a) transgenes were visualized using confocal microscopy (scale bars = 50 μm). The middle rows show digital enlargements of the regions boxed in the top rows (scale bars = 25 μm)

As the *pex5‐2* mutant presented more complete IBA resistance but less severe defects in processing PTS2 proteins than *pex5‐1*, we directly compared peroxisomal matrix protein import in these alleles by using confocal microscopy to visualize import of PTS1‐ and PTS2‐tagged GFP reporters (Woodward & Bartel, 2005a; Zolman & Bartel, 2004). Wild type showed the expected GFP‐PTS1 and PTS2‐GFP puncta in seedling cotyledons and hypocotyls (Figure 3a,b), indicating efficient PTS1 and PTS2 import. As previously reported (Woodward & Bartel, 2005a), *pex5‐1* displayed wild‐type‐like GFP‐PTS1 puncta (Figure 3a, Supporting Information Figure S3) coupled with largely cytosolic PTS2‐GFP fluorescence (Figure 3b, Supporting Information Figure S4). In contrast, *pex5‐2* showed a mixture of cytosolic and punctate GFP‐PTS1 fluorescence (Figure 3a, Supporting Information Figure S3) and punctate PTS2‐GFP fluorescence (Figure 3b, Supporting Information Figure S4). Quantification of punctate (peroxisomal) versus dispersed (cytosolic) fluorescence revealed that *pex5‐2* imported only a fraction (20%–50% in cotyledons; 5%–12% in hypocotyls) of GFP‐PTS1, whereas wild type and *pex5‐1* imported more than 90% of GFP‐PTS1 (Supporting Information Figure S3). Conversely, *pex5‐2* imported PTS2‐GFP at least as well as wild type (over 90%) compared to less than 15% PTS2‐GFP import for *pex5‐1* (Supporting Information Figure S4). Although PTS2‐GFP fluorescence appeared brighter in *pex5‐2* than wild type (Figure 3b), images collected at different gain settings revealed that peroxisomes were of similar sizes in *pex5‐2* and wild type (Supporting Information Figure S4a,b), and immunoblotting revealed more GFP in the *pex5‐2* line compared to wild type or *pex5‐1* (Supporting Information Figure S4c). As the transgene in *pex5‐2* was introduced by crossing from the wild‐type line, this expression difference is likely due to different degrees of silencing in the different lines. We concluded that the pex5‐2 lesion in the PTS1‐binding TPR domain (Figure 1) specifically impaired PTS1 import while leaving PTS2 import intact.

### 3.3 Elevated growth temperature exacerbates *pex5‐2* physiological and molecular defects

**Figure 4:**
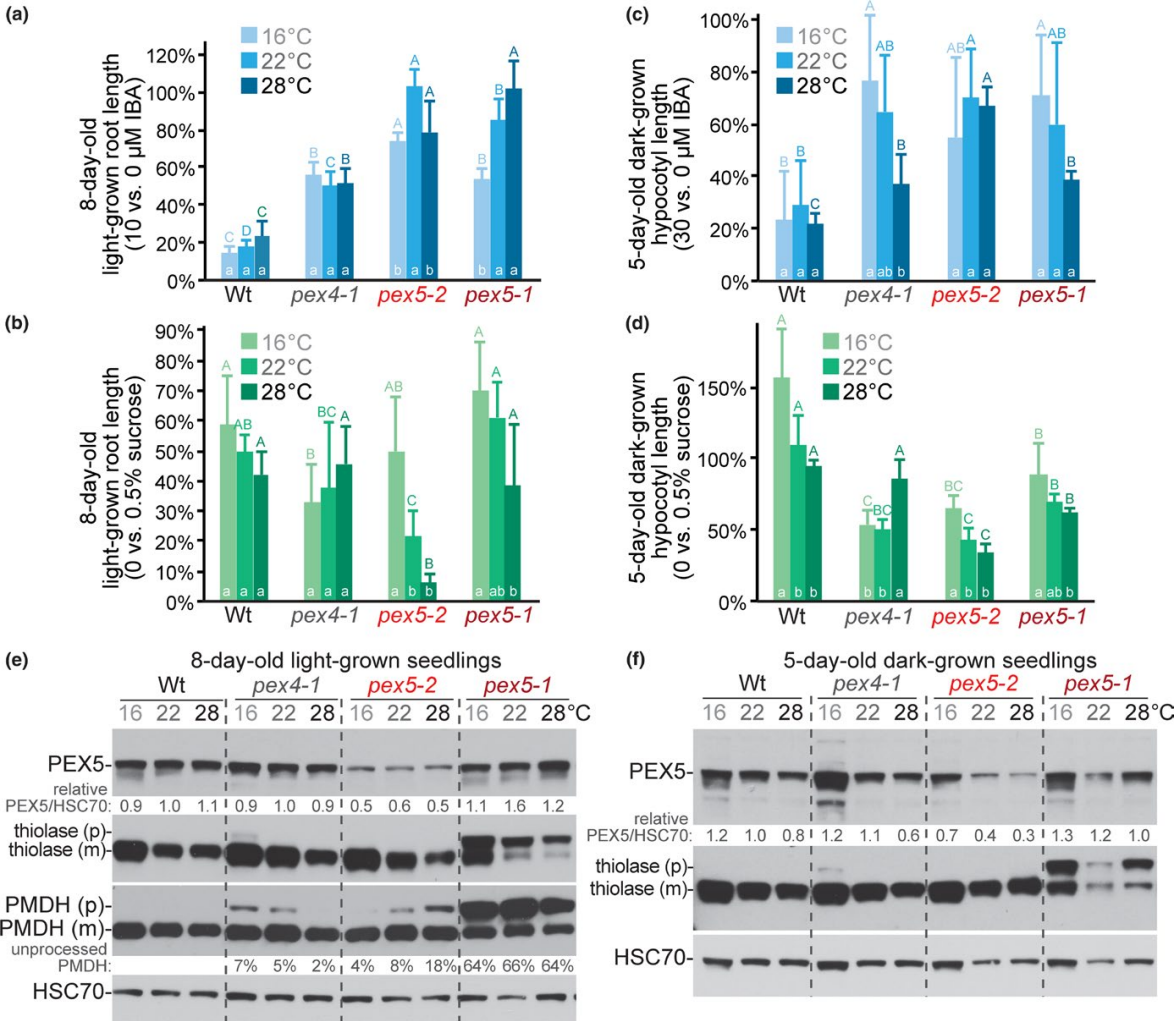
Growth at elevated temperature exacerbates some *pex5‐2* defects. (a–d) Seedlings were grown in the light (a, b) or the dark (c, d) on medium containing sucrose, containing sucrose and IBA (a, c), or lacking sucrose (b, d). Light‐grown root lengths (a, b) or dark‐grown hypocotyl lengths (c, d) were normalized to the corresponding mean length on sucrose‐supplemented medium. Bars show the means of three (28°C) or four (16°C and 22°C) biological replicates (each with ≥12 seedlings); error bars show standard deviations. Different lowercase letters within bars indicate significant differences within a genotype at different temperatures (one‐way ANOVA,* p *<* *0.05). Different uppercase letters above bars indicate significant differences between genotypes at the same temperature (one‐way ANOVA,* p *<* *0.05). (e, f) Extracts from seedlings grown at the indicated temperatures in the light (e) or dark (f) were processed for immunoblotting and probed with antibodies to the indicated proteins. PEX5 levels (quantified using ImageJ) were normalized to HSC70 and then to the 22°C Wt level (set at 1.0) to give the numbers below the PEX5 panels (means of three biological replicates, including the one shown). PMDH and thiolase are synthesized as precursor (p) proteins that are processed into mature forms (m) after peroxisome entry. The percentages of unprocessed PMDH (means of three biological replicates, including the one shown) in the mutants are shown below the PMDH panel. HSC70 was used to monitor loading

After importing PTS1 cargo, PEX5 is ubiquitinated via the peroxisomal ubiquitination machinery. PEX4 is a ubiquitin‐conjugating enzyme implicated in the ubiquitination that facilitates PEX5 retrotranslocation from the peroxisome membrane. The peroxisomal impairments of the *pex4‐1* mutant (Zolman et al., 2005) are less severe when seedlings are grown at elevated temperature in the dark (Kao & Bartel, 2015). Moreover, PEX5 levels are lower in dark‐grown seedlings at elevated growth temperature (Kao & Bartel, 2015). These results suggest the possibility that accumulated PEX5 protein contributes to *pex4‐1* physiological defects. To examine whether growth temperature also impacted *pex5‐2* phenotypes, we compared growth at three temperatures. In light‐grown seedlings, *pex5‐2* roots were highly IBA resistant when grown at all tested temperatures (16, 22, or 28°C) (Figure 4a), whereas *pex5‐2* root growth without sucrose was impaired at the normal growth temperature (22°C), further impaired at 28°C, but wild type‐like at 16°C (Figure 4b). Similar to light‐grown roots, *pex5‐2* dark‐grown hypocotyls were highly IBA resistant regardless of growth temperature (Figure 4c), whereas *pex5‐2* hypocotyl growth without sucrose was most impaired at high temperature (Figure 4d). Moreover, growth at 28°C resulted in slight accumulation of the precursor form of PMDH in light‐grown *pex5‐2* seedlings (Figure 4e), suggesting worsened PTS2 processing at higher temperature, whereas PMDH processing was nearly complete in *pex5‐2* seedlings grown at 16°C (Figure 4e), suggesting improved PTS2 processing at low temperature. In contrast to *pex5‐2*, PMDH processing in *pex4‐1* was improved at higher temperature and exacerbated at lower temperature (Figure 4e). Temperature seemed to have a less severe impact on *pex5‐1* than on *pex5‐2*. For example, PTS2 processing of PMDH was unchanged in *pex5‐1* grown at various temperatures (Figure 4e).

We also examined PEX5 levels following growth at these three temperatures. Interestingly, the general decline in PEX5 levels that accompanied higher growth temperature in dark‐grown wild‐type seedlings (Figure 4f; Kao & Bartel, 2015) was not observed in light‐grown seedlings (Figure 4e). *pex5‐2* seedlings generally accumulated less PEX5 protein than wild type at all temperatures tested (Figure 4e,f), suggesting that the worsened physiological (Figure 4b) and PTS2‐processing defects (Figure 4e) of *pex5‐2* at high temperature did not stem from a magnified decrease in overall pex5‐2 protein level at high temperature.

## 4 DISCUSSION

### 4.1 A novel *pex5* allele displaying PTS1‐specific defects

Most Arabidopsis peroxisomal matrix proteins carry a PTS1 (Reumann, 2004) and are delivered to peroxisomes via the PTS1 receptor PEX5. One of the first reported Arabidopsis peroxin mutants carried a *PEX5* missense mutation (Zolman et al., 2000). This allele, *pex5‐1*, has normal PTS1 import coupled with substantial PTS2 import defects (Figure 3; Woodward & Bartel, 2005a), presumably because the *pex5‐1* mutation disrupts a predicted PEX7‐binding region (Figure 1a). Subsequent analysis of a T‐DNA insertional mutant, *pex5‐10* (Zolman et al., 2005), revealed both PTS1 and PTS2 import defects (Khan & Zolman, 2010; Lingard & Bartel, 2009) due to an internal deletion in the PEX5 protein that includes several predicted PEX14‐binding domains (Figure 1a; Khan & Zolman, 2010). Similarly, expressing a truncated pex5 protein (1–657 aa) that lacks TPR7 and the C‐terminal region in wild type or reducing PEX5 levels using RNAi confers both PTS1 and PTS2 import defects (Hayashi et al., 2005). In this work, two decades after the first forward genetic screens for Arabidopsis seedlings with peroxisome‐related defects (Hayashi et al., 1998; Zolman et al., 2000), we report the first *pex5* mutant specifically impaired in PTS1 import. In addition to inefficient PTS1 import (Figure 3, Supporting Information Figure S3), the *pex5‐2* mutant displayed typical peroxisome defects, including poor growth without sucrose supplementation (Figure 4b,d), resistance to IBA‐mediated inhibition of root and hypocotyl elongation (Figures 2a, 4a,c), and slight accumulation of an unprocessed PTS2 protein (Figures 2b and 4e).

The Gly498Arg substitution in *pex5‐2* is in the second of seven TPR domains in the PEX5 C‐terminal region (Figure 1). Although examination of the crystal structure of the human PEX5 TPR domain (Stanley et al., 2006) suggests that the affected Gly residue does not directly interact with the PTS1 of the cargo protein (Figure 1c), this residue is conserved in PEX5 orthologs in metazoans, trypanosomes, and fungi (Figure 1b). PTS1 import was substantially impaired in the *pex5‐2* mutant (Figure 3a), suggesting that the Gly‐to‐Arg substitution might impede folding of TPR2 or the TPR domain in general, thus reducing PTS1 binding.

Although the PTS2 pathway is absent in fruit flies (Faust, Verma, Peng, & McNew, 2012), nematodes (Gurvitz et al., 2000; Motley, Hettema, Ketting, Plasterk, & Tabak, 2000), and diatoms (Gonzalez et al., 2011), plant peroxisomes use both PTS1 and PTS2 pathways. Given the reciprocal GFP‐PTS1 and PTS2‐GFP import defects in *pex5‐2* and *pex5‐1* (Figure 3), comparing the phenotypes of these mutants can start to illuminate the relative importance of PTS1 versus PTS2 import. For example, both *pex5‐1* and *pex5‐2* exhibit IBA resistance (Figures 2a, 4a,c) and growth defects that can be ameliorated by sucrose (Figure 4b,d), suggesting that β‐oxidation requires both PTS1 and PTS2 enzymes. Although all of the enzymes implicated exclusively in IBA β‐oxidation are PTS1 proteins (Strader et al., 2011; Zolman, Martinez, Millius, Adham, & Bartel, 2008; Zolman, Nyberg, & Bartel, 2007), the 3‐ketoacyl‐CoA thiolase catalyzing the final step of both IBA and fatty acid β‐oxidation (Germain et al., 2001; Hayashi et al., 1998; Strader & Bartel, 2011) is a PTS2 protein. Moreover, the acyl‐CoA oxidases acting early in fatty acid β‐oxidation include both PTS1‐ and PTS2‐containing isozymes (Adham, Zolman, Millius, & Bartel, 2005). In contrast to shared β‐oxidation defects, we observe shoot growth defects in *pex5‐2* but not *pex5‐1* (Figure 2c), perhaps because the photorespiration enzymes glycolate oxidase, Ser:glyoxylate aminotransferase, glyoxylate:Glu aminotransferase, and hydroxypyruvate reductase are all PTS1 proteins (Fukao, Hayashi, & Nishimura, 2002) that we predict would be efficiently imported into *pex5‐1* but not *pex5‐2* peroxisomes.

### 4.2 Genetic and environmental controls of PEX5 levels and function

Growth temperature can influence peroxisome function in *pex* mutants. For example, reduced growth temperature improves import of catalase, a PTS1 protein, in mammalian cell lines carrying mutations found in several groups of peroxisome biogenesis disorder patients (Fujiwara et al., 2000; Imamura, Tamura, et al., 1998; Imamura, Tsukamoto, et al., 1998). In contrast, growth at elevated temperature decreases PEX5 levels and improves growth, IBA responsiveness, and PTS2 processing in dark‐grown *pex4* seedlings (Figure 4f; Kao & Bartel, 2015). The effects of temperature on *pex4* defects suggest that the detrimental impact of excess PEX5 in the peroxisomal membrane when PEX4 is dysfunctional is partially relieved by decreased overall PEX5 levels at elevated temperature (Kao & Bartel, 2015).

Seedling pex5‐2 protein levels were slightly lower than pex5‐1 or wild‐type PEX5 protein levels (Figure 4e,f). Decreased pex5‐2 protein levels could reflect a TPR‐folding defect that increases PEX5 susceptibility to degradation. Alternatively or in addition, PTS1 cargo binding, which we expect to be impaired by the *pex5‐2* mutation, might protect PEX5 from degradation. Low growth temperature slightly increased pex5‐2 protein levels in dark‐grown seedlings but not light‐grown seedlings (Figure 4e,f). However, because we observed the most dramatic impacts of temperature on *pex5‐2* physiological and molecular defects in light‐grown seedlings (Figure 4b,e), it seems likely that *pex5‐2* defects stem more directly from altered pex5‐2 function caused by the Gly498Arg change and not solely from decreased pex5‐2 levels.

In addition to *pex5‐2*, other Arabidopsis mutants with low PEX5 levels include *pex6* and *pex26* mutants (Gonzalez et al., 2017; Zolman & Bartel, 2004), which show defects in retrotranslocating PEX5 from the peroxisomal membrane (Gonzalez et al., 2017; Ratzel et al., 2011), and *pex7* mutants (Ramón & Bartel, 2010), which are defective in the PEX5‐interacting PTS2 receptor and show import defects of not only PTS2 proteins but also PTS1 proteins (Ramón & Bartel, 2010; Woodward & Bartel, 2005a). Interestingly, reducing function of PEX2, one of the peroxisomal RING ubiquitin‐protein ligases, restores PEX5 levels in *pex6‐1* and *pex26‐1* but not *pex7‐1* mutants (Burkhart et al., 2014; Gonzalez et al., 2017), suggesting multiple avenues for PEX5 degradation. It will be interesting to learn whether the apparent pex5‐2 instability that we observe can be attributed to the peroxisomal ubiquitination machinery.

When PEX5 retrotranslocation is inefficient, as in mammalian *pex1* mutants, PEX5 ubiquitination is associated with peroxisome degradation via specialized autophagy (Law et al., 2017). Similarly, genetically preventing autophagy improves peroxisome function in Arabidopsis *pex1* and *pex6* mutants (Gonzalez et al., 2018; Rinaldi et al., 2017). The allelic series of *pex5* mutants may be useful in future dissection of the possible role of PEX5 in promoting autophagy of peroxisomes (pexophagy) in plants.

### 4.3 Consequences and causes of PTS1‐specific import defects

One hallmark of *pex* mutants is reduced PTS2 processing (reviewed in Bartel et al., 2014), which can vary in severity in different tissues or ages (Kao et al., 2016). The protease DEG15 cleaves the N‐terminal PTS2‐containing region (Helm et al., 2007; Schuhmann et al., 2008). As DEG15 is a PTS1 protein, PTS2 processing is expected to require robust PTS1 and PTS2 import. Interestingly, however, the strong PTS1 import defect observed in *pex5‐2* (Figure 3a) was accompanied by only minimal PTS2‐processing defects (Figures 2b, 4e,f), suggesting that a small amount of DEG15 import is sufficient for substantial PTS2 processing and that more severe PTS2‐processing defects probably reflect primarily PTS2 import defects. Of course, the ability to detect PTS2‐processing defects relies on the stability of the precursor protein in the cytosol, which might vary for different proteins. For example, thiolase precursor levels increase in *pex* seedlings treated with the MG132 proteasome inhibitor (Kao & Bartel, 2015), implying that cytosolic thiolase is susceptible to ubiquitin‐dependent degradation. Differences in cytosolic precursor stability could contribute to the apparent differences in PMDH and thiolase‐processing efficiencies in *pex5‐2* (Figures 2b and 4e) and other *pex* mutants (Burkhart et al., 2014; Gonzalez et al., 2017, 2018; Kao et al., 2016; Monroe‐Augustus et al., 2011).

In addition to *pex5‐2*, several other Arabidopsis *pex* mutants display impaired PTS1 import but apparently normal PTS2 import. The *pex2‐1*,* pex4‐1*, and *pex10‐2* mutants, which are defective in the PEX5‐recycling ubiquitination machinery, display punctate PTS2‐GFP and a mixture of cytosolic and punctate GFP‐PTS1 (Burkhart et al., 2014). These receptor‐recycling mutants have slightly elevated PEX5 levels (Kao et al., 2016), and *pex4‐1* exhibits increased PEX5 membrane association (Kao & Bartel, 2015; Ratzel et al., 2011), as expected when PEX5 recycling from the peroxisomal membrane is impaired. Both peroxisomal membrane‐associated PEX5 and insufficient PEX5‐PTS1 recognition are detrimental to PTS1 import, indicating that the localization and quantity of PEX5 are important to maintain peroxisome function.

Salt (NaCl) increases Arabidopsis peroxisome abundance (Fahy et al., 2017; Frick & Strader, 2018; Mitsuya et al., 2010) and enhances the inhibitory effect of IBA (Supporting Information Figure S1a), presumably because increased numbers of peroxisomes host more β‐oxidation. Although *pex5‐2* was selected in a screen for salinity‐related peroxisome proliferation factors, subsequent testing revealed that salt still increased IBA responsiveness in *pex5‐2* (Supporting Information Figure S1a), suggesting that the peroxisome proliferation machinery remained functional and that PTS1 import is not necessary for this response. In contrast, salt did not similarly improve IBA responsiveness in *pex6‐1* roots (Supporting Information Figure S1a), hinting that PEX6 may be involved in salt‐induced peroxisome proliferation. Future identification of mutants that do not increase IBA responsiveness in response to salt treatment might identify additional components of this response.

## 5 CONCLUSIONS

The metabolic activities compartmentalized in Arabidopsis peroxisomes allow quantification of peroxisome function and dysfunction in an intact multicellular organism. The *pex5‐2* mutant described here provides insights into peroxisomal matrix protein import and the relationship between PTS1 import and PTS2 protein processing. With its PTS1‐specific defects, the *pex5‐2* mutant extends the allelic series that includes the PTS2‐specific *pex5‐1* (Woodward & Bartel, 2005a; Zolman et al., 2000) and the general import defective *pex5‐10* (Khan & Zolman, 2010; Zolman et al., 2005), providing a valuable asset for future peroxisome research.

## AUTHOR CONTRIBUTIONS

YTK and BB designed the research; YTK and KJP conducted the mutant screen; KJP performed the physiological and molecular characterizations; RJL, YTK, and KJP performed the microscopy; YTK, KJP, and BB wrote the manuscript; all authors revised the manuscript and approved the final version.
