# Supplementary material for: A PEX5 missense allele preferentially disrupts PTS1 cargo import into Arabidopsis peroxisomes
Source: Plant Direct. 2019 Mar 20;3(3):e00128. doi: 10.1002/pld3.128 (PMC6508846; doi:10.1002/pld3.128)
Supplement: Supplementary file 1 [file PLD3-3-e00128-s001.pdf]

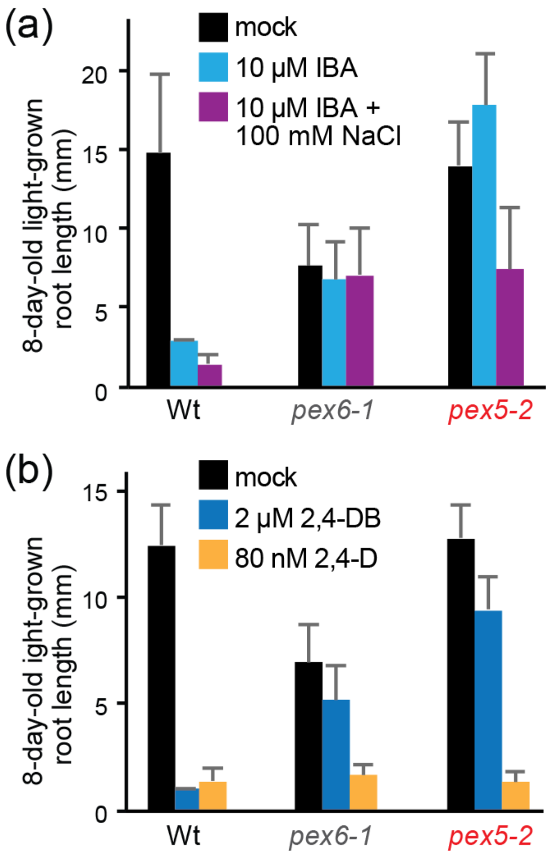

Supporting Information Figure S1. *pex5-2* displays resistance to IBA and the synthetic IBA analog 2,4-dichlorophenoxybutyric acid (2,4-DB) but not the synthetic auxin analog 2,4-dichlorophenoxyacetic acid (2,4-D).

(a) *pex5-2* is resistant to the inhibitory effects of IBA on root elongation. Bars indicate mean root lengths of 8-day-old seedlings grown in light on media supplemented with 0.5% sucrose in the absence or presence of 10 µM IBA or 10 µM IBA and 100 mM NaCl ($n \geq 8$, except for Wt on 10 µM IBA + 100 mM NaCl, where only 4 of 13 seeds germinated).

(b) *pex5-2* is resistant to the inhibitory effects of 2,4-DB on root elongation but responds similarly to wild type to the synthetic auxin 2,4-D. Bars indicate mean root lengths of 8-day-old light-grown seedlings grown on media supplemented with 0.5% sucrose in the absence or presence of 2 µM 2,4-DB or 80 nM 2,4-D ($n \geq 12$).

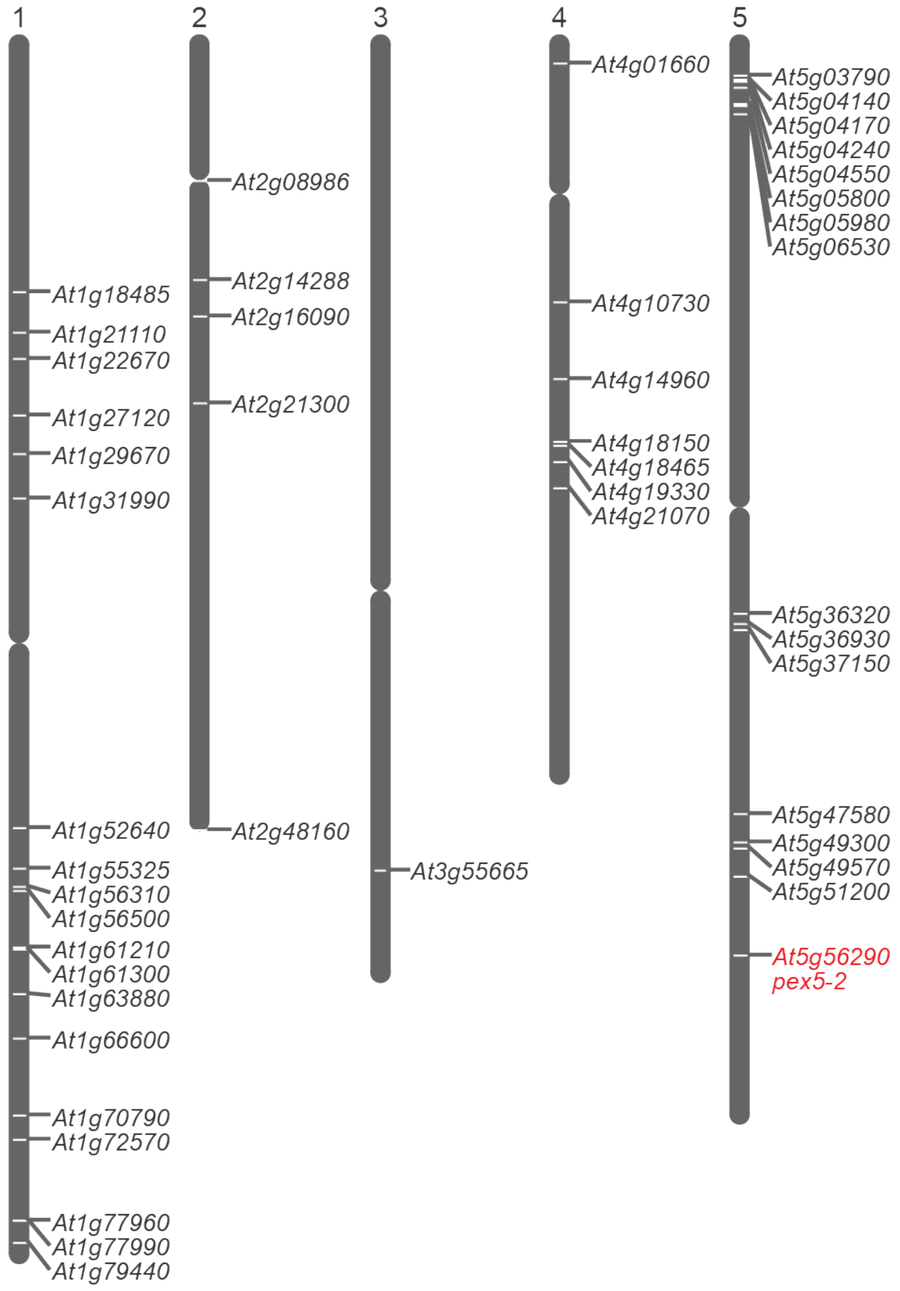

Supporting Information Figure S2. Whole-genome sequencing revealed the *pex5-2* mutation. Genomic DNA from pooled M_3 seedlings was sequenced and examined for EMS-consistent single-nucleotide changes (G/C to A/T) that differed from our lab strain of Col-0. Homozygous non-synonymous mutations in coding regions or splice sites are indicated by gene identifier numbers next to the five Arabidopsis chromosomes. The map was generated using the Arabidopsis Information Resource Center Chromosome Map tool.

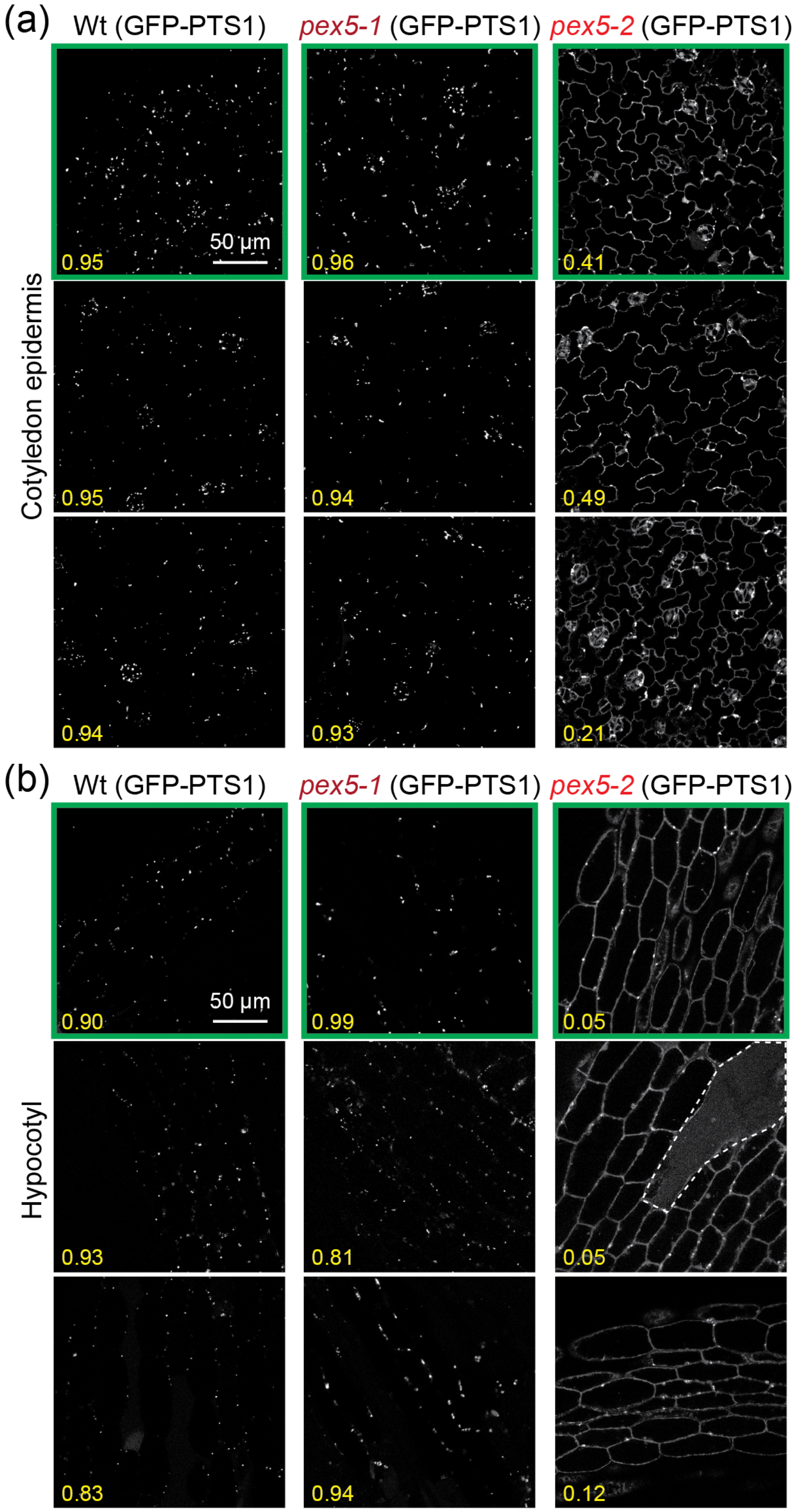

Supporting Information Figure S3. *pex5-2* displays defective GFP-PTS1 import. Cotyledon epidermal (a) and hypocotyl cells (b) of 4-day-old light-grown seedlings carrying the *35S:GFP-PTS1* (Zolman and Bartel, 2004) transgene were visualized using confocal microscopy with a 40x objective. GFP fluorescence (white) was collected between 493 and 552 nm. Each image corresponds to a 1.0-µm optical section (pinhole = 1 airy unit) and is an average of 4 exposures. Scale bars = 50 µm. Relative GFP-PTS1 import (the fraction of punctate/total fluorescence determined using ImageJ) is shown in lower left corners. The area enclosed in the dashed white line was masked prior to quantification for the middle *pex5-2* hypocotyl image. Digital enlargements of the images boxed in green were used to generate Figure 3a.

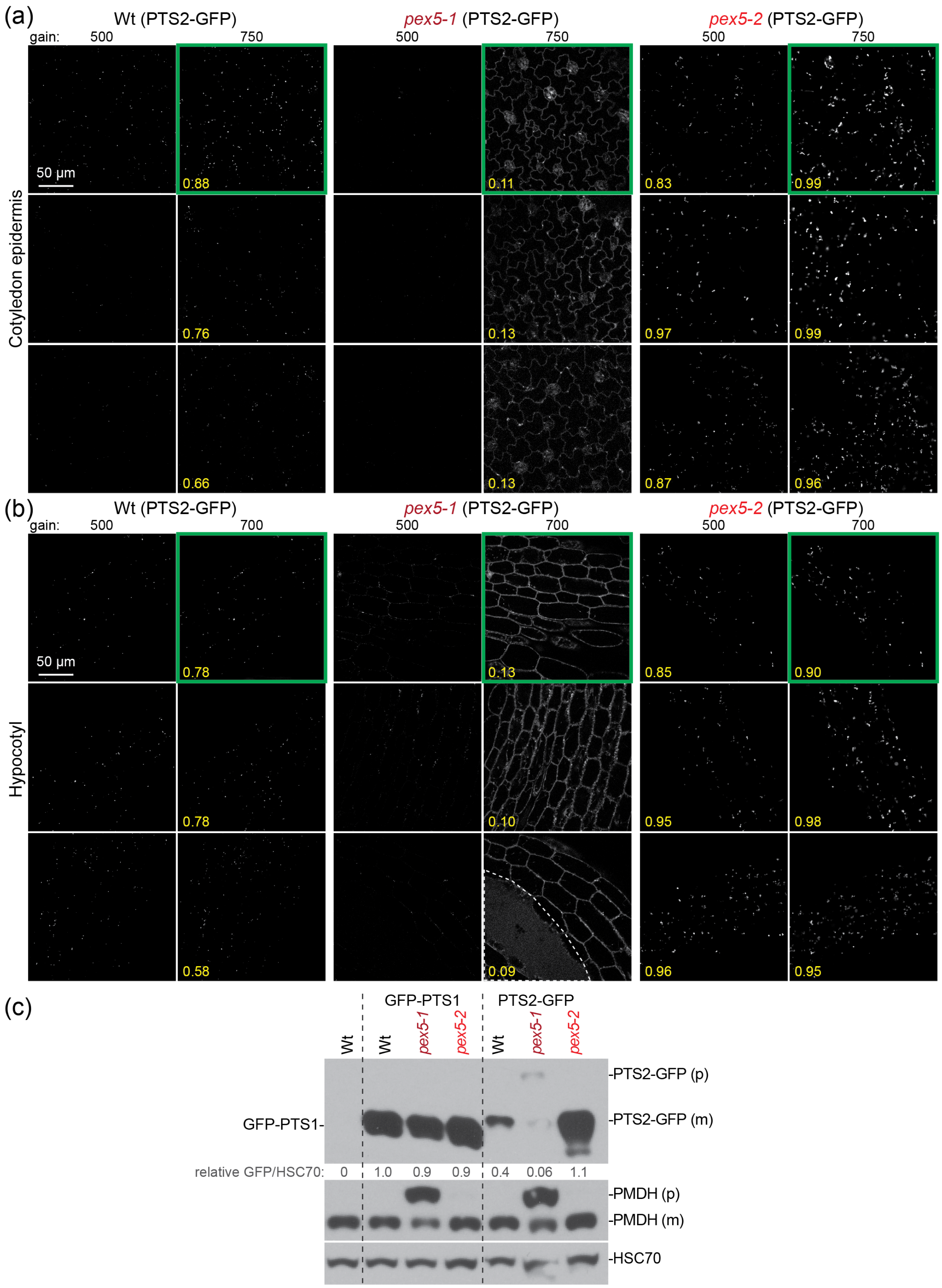

Supporting Information Figure S4. PTS2-GFP import resembles wild type in *pex5-2*.
(a, b) Cotyledon epidermal (a) and hypocotyl cells (b) of 4-day-old light-grown seedlings

carrying the *35S:PTS2-GFP* (Woodward and Bartel, 2005a) transgene were visualized using confocal microscopy with a 40x objective. GFP fluorescence (white) was collected between 493 and 552 nm. Each image corresponds to a 1.0-μm optical section (pinhole = 1 airy unit) and is an average of 4 exposures. Scale bars = 50 μm. Sequential images from the same field were collected using two gain settings to allow comparison of peroxisomes in wild type and *pex5-2*, which displayed brighter fluorescence due to elevated GFP levels (panel c). Relative PTS2-GFP import (the fraction of punctate/total fluorescence determined using ImageJ) is shown in lower left corners. The area enclosed in the dashed line was masked prior to quantification for the bottom *pex5-1* hypocotyl image. Digital enlargements of the images boxed in green were used to generate Figure 3b.

(c) GFP protein levels are higher in *pex5-2* (PTS2-GFP) compared to wild type (PTS2-GFP). Extracts from 7-day-old light-grown seedlings were processed for immunoblotting and probed with antibodies to the indicated proteins. GFP protein levels (quantified using ImageJ) were normalized to HSC70 and then to the Wt (GFP-PTS1) level (set at 1.0) to give the relative GFP/HSC70 levels shown below the GFP panel.

Supporting Information Table S1: PCR-based genotyping markers

Mutation or transgene (Reference)	Primers (5' to 3')	Restriction enzyme	Product size (bp) Wt	Product size (bp) Mutant or transgene
35S:PEX5 (Zolman and Bartel, 2004)	PEX5-38 (TGAAGACCAACAGATAAGG)<br>PEX5-39 (CCCATTGGAGGCATAGG)	-	264	168
35S:GFP-PTS1 (Zolman and Bartel, 2004)	35S-F (GGATGACGCACAATCCCACTATCCTTCG)<br>GFP-1 (TTGAAAAGCATTGAACACCATAAGAGAAAGT)	-	-	321
35S:PTS2-GFP (Woodward and Bartel, 2005a)	35S-F (GGATGACGCACAATCCCACTATCCTTCG)<br>GFP-1 (TTGAAAAGCATTGAACACCATAAGAGAAAGT)	-	-	476
pex4-1 (Zolman et al., 2005)	PEX4-A (TGCATCTCTTTTTATAACAACCTTCTCC)<br>PEX4-B (GAACTAGAACCGAACGGGAACCAAACC)	MnlI	201, 91	292
pex5-1 (Zolman et al., 2000)	PEX5-B (TCATCAATAATAAGTTCACCACGGCTCATCT)<br>PEX5-32 (GTTGGACGACATATCTCTTTTCTGG)	EcoRI	117, 59, 77	176, 77
pex5-2 (this work)	PEX5-5 (TGAGAATGCTGAAGGTTGGAGATTACTT)<br>PEX5-6 (GCACGCATCATTGCAGCTATTGCCTGCAT)	DdeI	123, 61	97, 26 61
pex5-10 T-DNA (Zolman et al., 2005)	LB1-SALK (CAAACCAGCGTGGACCGCTTGCTGCAACTC)<br>PEX5-21 (GATATCAAATGCGACTCAAACACTGATGAC)	-	-	405
Wild-type *PEX5* (Zolman et al., 2005)	PEX5-3 (GTCGTTGGCTGAATATTTTGTTCGGC)<br>PEX5-21 (GATATCAAATGCGACTCAAACACTGATGAC)	-	541	-
pex6-1 (Zolman and Bartel, 2004)	F10O3-7 (CAGACTTTACTGGCAAAAGCTGTGGCG)<br>F10O3-T (GCTTGCACCTATAATAAACAGATCCTGGG)	XhoI	270, 115	385

Supporting Information Macro 1: ImageJ macro for measuring total intensity

(Excludes 1-pixel background)

```
setOption("BlackBackground", false);
run("Make Binary");
run("Set Measurements...", "min integrated display redirect=None decimal=3");
run("Analyze Particles...", "size=2-Infinity pixel circularity=0-1.00 show=[Bare Outlines] clear
    add");
close();
run("Revert");
roiManager("Measure");
```

Supporting Information Macro 2: ImageJ macro for measuring puncta intensity

(Captures puncta of at least 15 pixel diameter and between 0.2-1.0 circularity)

```
setOption("BlackBackground", false);
run("Make Binary");
run("Set Measurements...", "min integrated display redirect=None decimal=3");
run("Analyze Particles...", "size=15-Infinity pixel circularity=0.20-1.00 show=[Bare Outlines] clear
    add");
close();
run("Revert");
roiManager("Measure");
```
