# Supplementary material for: A PEX5 missense allele preferentially disrupts PTS1 cargo import into Arabidopsis peroxisomes
Source: Plant Direct. 2019 Mar 20;3(3):e00128. doi: 10.1002/pld3.128 (PMC6508846; doi:10.1002/pld3.128)
Supplement: Supplementary file 2 [file PLD3-3-e00128-s002.pdf]

Response to reviewers
We appreciate the care with which the reviewers read our manuscript and the useful suggestions that were provided for improvement. We address each reviewer comment below and have provided corresponding clarifications and additions to the revised text and figures.

Reviewer #1:
The manuscript by Patel et al describe a new allele of PEX5 with defects in PTS1 but not PTS2 import to peroxisomes. This allele could become a unique tool for future characterization of peroxisome biogenesis. The results clearly show a distinct phenotype to other pex5 mutants.

While the experiments are in principle sound, there are some instances were quantification of a phenotype is warranted to conclude a particular effect of the pex5-2 mutation. These follow-up analyses could be done with data already acquired, and are needed to validate important conclusions of the paper. In addition, there are several instances of over-interpretation that need to be addressed before the paper can be published. A minor (but significant) issue, however, is the writing, since there are several paragraphs that need lots of editing to make the article clear.

Core issues:
- Figure 3: "In contrast, pex5-2 showed a mixture of cytosolic and punctate GFP-PTS1 fluorescence (Figure 3a) and punctate PTS2-GFP fluorescence without appreciable cytosolic signal (Figure 3b)." Please include insets for these images with higher magnification to clearly show the cytosolic signal in pex5-2 when compared to WT. Higher mag images will also discard the possibility that size of peroxisomes is affected in the mutant.

Response: As suggested, we added higher magnification images of the cotyledon images in this figure to more clearly show the peroxisome versus cytosolic signals.

In the case of pex5-2 with PST2-GFP, while the authors are correct that the marker is transported into the peroxisome, the apparent morphology and size seems to be very different than the WT. I don't think you can conclude from this data that PTS2 import is "intact". Since abnormal import of PTS2 proteins could result in altered biogenesis of this organelle, this should be clarified. Is there a peroxisomal marker that the authors can use to confirm that these indeed peroxisomes?

Response: We also noticed that peroxisomes appeared brighter with the PTS2-GFP line in *pex5-2* compared to wild type. However, we do not believe that the peroxisomes are bigger in *pex5-2* than in wild type. First, the GFP-PTS1 marker does detect some puncta in *pex5-2* that appear wild type in size (e.g., Figure 3a). Moreover, when we capture *pex5-2* (PTS2-GFP) images at a lower gain, they appear to be similarly sized as wild-type (PTS2-GFP) peroxisomes imaged at the higher gain settings (new supplemental Figure 4c). Finally, we examine GFP levels in these lines by using immunoblotting, and we detected more GFP in the *pex5-2* (PTS2-GFP) line than the wild type (PTS2-GFP) (new supplemental Figure 4c), consistent with the brighter fluorescence in this line. Because we generated the *pex5-2* (PTS2-GFP) by crossing to the wild type (PTS2-GFP) line, these differences are not due to the position of the transgene in the genome, but rather suggest that the *35S:PTS2-GFP* transgene is partially silenced in our wild-type line. We have included the GFP immunoblot and images taken at different gain settings in the new supplemental Figure 4c to illustrate these findings for the reader, and have modified the text accordingly.

In addition, we quantified import in these experiments using ImageJ and added the macros that we used for this quantification in the supporting information. We calculated the intensity in

puncta (peroxisomes – defined as at least 15 adjacent pixels with a circularity of 0.2-1.0) and the total intensity in the image (defined as at least 2 adjacent pixels without regard to circularity) and present the ratio (puncta/total) as fractional import. This quantification is now included in the new supplemental figures S3 and S4. This quantification validates the overall conclusion that GFP-PTS1 import (but not PTS2-GFP import) is impaired in *pex5-2*, which is also qualitatively apparent in the representative images shown.

"Because the pex5-2 mutant showed decreased PEX5 levels when grown at normal temperature," I do not see evidence showing the levels of PEX5 in pex5-2. If anything, this statement contradicts Fig 2 where pex5-2 seems to make similar levels of PEX5. The authors should quantify the bands in Fig 2b if a statement about abundance is to be made.

Response: As suggested, we have removed the mention of PEX5 levels in Figure 2 from the rationale for the temperature experiment (which is now clarified as well). We have added quantification of relative PEX5 levels (from three biological replicates of this experiment) in the various genotypes and growth conditions in Figure 4 below the PEX5 panel.

"In contrast, the mixture of punctate and cytosolic GFP-PTS1 observed in pex5-2 grown at 16 or 22°C appeared to be more cytosolic at 28°C (Figure 5)". Again, a statement of increased cytosolic accumulation of this marker needs some quantification and needs to be derived from several images and multiple experiments. You could, for example, count number of peroxisomes per area unit (using always similar medial sections) and normalize by the avg intensity of the "cytosolic signal".

Response: As suggested, we quantified import in these experiments using ImageJ and we agree with the reviewer that the temperature effects on import, which are confounded by the increased cell size at higher temperature, are not sufficiently convincing to include in the manuscript, so we removed Figure 5.

Over-interpretation:
In the abstract: "Experiments decreasing or increasing growth temperatures ameliorated or exacerbated of pex5-2 peroxisome-related defects, respectively," I don't think this is accurate. The experiment tested growth at three different temperatures, but they did not "increase or decrease" temperature. This would mean that plants were moved from high to low or low to high temps.

Response: We agree that this wording was confusing and have reworded this sentence to "Growth at reduced or elevated temperatures ameliorated or exacerbated pex5-2 peroxisome-related defects, respectively…"

Results: "As with IBA responsiveness (Figure 2a), expressing wild-type PEX5 in the pex5-2 mutant improved rosette size in the mutant (Figure 2c)," include here that while rosette size was better than the mutant, the 35S construct did not fully complement the shoot phenotype. Plants are clearly small.

Response: We also were puzzled that the *35S:PEX5* construct fully rescued *pex5-2* seedling phenotypes but did not fully rescue *pex5-2* adult growth defects. To explore the incomplete rescue of the older *pex5-2* plants, we performed immunoblotting of leaf tissue from 5-week-old plants of the genotypes shown in Figure 2. Surprisingly, while we were able to detect the wild-type PEX5 or the mutant pex5 protein in the wild type and the single mutants, we did not detect PEX5 protein in either wild type or *pex5-2* carrying the *35S:PEX5* construct. We conclude that

this construct, which clearly leads to PEX5 accumulation and rescues *pex5-2* in seedlings (Figure 2a and 2b), is causing co-suppression in adult plants. Therefore, although the *pex5-2* adult growth defects persist after backcrossing, we cannot definitively conclude that the *pex5-2* growth defects visible in 5-week-old plants are caused by the *pex5-2* mutation, and we have removed the adult plant photo from Figure 2c.

Needs to improve writing:
Results: "Thus IBA treatment confers high auxin phenotypes including reduced root and hypocotyl elongation in wild-type seedlings" The transition here is odd, it seems like you are missing a statement for this phenotype being dependent on peroxisomes?

Response: We have clarified this and the previous sentence to read: “Thus in seedlings with functioning peroxisomes, IBA treatment confers high-auxin phenotypes including reduced root and hypocotyl elongation (Zolman et al., 2000; Strader et al., 2011), and IBA-resistance screens have uncovered genes needed for peroxisome biogenesis and function (reviewed in Bartel et al., 2014).”

Same section "We isolated seedlings with elongated roots on normally inhibitory concentrations of IBA and NaCl." Describe the rationale here with more detail. Again, there is something implied that it is not obvious to the reader and makes it difficult to follow.

Response: We clarified the first paragraph of the results to more clearly describe our screening rationale and outcome: “Because salt increases Arabidopsis peroxisome abundance (Mitsuya et al., 2010; Fahy et al., 2017; Frick and Strader, 2018), we reasoned that screening for IBA resistance in the presence of salt might uncover factors impacting salinity-induced peroxisome proliferation. We therefore identified seedlings with elongated roots on normally inhibitory concentrations of IBA and NaCl.”

"The pex5-2 mutation results in a Gly498Arg substitution in one of the seven tetratricopeptide repeat (TPR) domains (Figure 1) that comprise the PTS1-binding region (Terlecky et al., 1995; Gatto et al., 2000)." You could describe Fig1c here. The 3D model is not mentioned at all until the discussion section.

Response: As suggested, we moved the initial description of Figure 1c from the discussion to the results section.

The description of Figure 4 needs a lot of work. The text jumps from a to b and then compares b vs d, and this is all really confusing. Figures should be described as a, then b then c then d. Again, lots of ideas seem to be implied but not stated. Some examples:
" the pex4-1 mutant (Zolman et al., 2005), which is defective in a ubiquitin-conjugating enzyme implicated in the PEX5 ubiquitination that facilitates retrotranslocation, displays less severe peroxisomal impairments when grown at elevated temperature in the dark" This is hard to understand. Something here is implied but not stated. I think you imply that PEX4 may ubiquitinate PEX5 at high temperature (?).

Response: We apologize for our lack of clarity in this section. As suggested, we have completely reworked the Figure 4 description in the results section to discuss the panels in alphabetical order and to more clearly describe both our rationale and our results.

"Indeed, we saw generally opposite effects of elevated temperature in pex5-2 compared to pex4-1" It is not clear what you mean by opposite effects. I see no change in root growth

between low and high temp for pex4 mutants in fig 4a. What is the opposite of "no change? Point exactly at the figure where you want the reader to focus on.

Response: As suggested, we have removed this sentence and instead explicitly compared each aspect of the *pex5-2* and *pex4-1* phenotypes.

"Growth at 28°C rather than the normal 22°C decreased pex5-2 root and hypocotyl elongation without sucrose supplementation (Figure 4b and 4d)" I could not understand this sentence at all.

Response: We clarified and expanded this explanation to read "In light-grown seedlings, *pex5-2* roots were highly IBA resistant when grown at all tested temperatures (16, 22, or 28°C) (Figure 4a), whereas *pex5-2* root growth without sucrose was impaired at the normal growth temperature (22°C), was further impaired at 28°C, but resembled wild type at 16°C (Figure 4b). Similar to light-grown roots, *pex5-2* dark-grown hypocotyls were highly IBA resistant regardless of growth temperature (Figure 4c), whereas *pex5-2* hypocotyl growth without sucrose was most impaired at high temperature (Figure 4d)."

Reviewer #2:
Patel et al. described a peroxisome-targeting signal (PTS) type signal1 (PTS1) receptor peroxin PEX5 Arabidopsis pex5-2 mutant with an exclusive PTS1 import defect. They also found that pex5-2 mutant shows typical peroxisome-related defects, including inefficient β-oxidation and reduced growth. Moreover, they claimed that pex5-2 mutation contributes to ameliorated or exacerbated peroxisome-related defects upon increment or decrement of growth temperatures. Overall, while I found that the discovery of functions related to pex5-2 mutation is interesting, I feel that some aspects of experimental results need to be strengthened to support their conclusions and more explanation on the result and discussion.

1. In Figure 2 (c), the growth defect in pex5-2 mutant is not fully complemented as can be seen for pex5-2/35S:PEX5 line. Since the complementation pex5-2/35S:PEX5 line did not restore to the wild-type phenotype. It is not very convincing to use that material to claim the growth defects were caused due to pex5-2 mutation. Authors need to have more explanation to support their results.

Response: Reviewer 1 shared this concern. As we indicate in our response to reviewer 1 above, we also were puzzled that the *35S:PEX5* construct fully rescued *pex5-2* seedling phenotypes but did not fully rescue *pex5-2* adult growth defects. To explore the incomplete rescue of the older *pex5-2* plants, we performed immunoblotting of leaf tissue from 5-week-old plants of the genotypes shown in Figure 2. Surprisingly, while we were able to detect the wild-type PEX5 or the mutant pex5 protein in the wild type and the single mutants, we did not detect PEX5 protein in either wild type or *pex5-2* carrying the *35S:PEX5* construct. We conclude that this construct, which clearly leads to PEX5 accumulation and rescues *pex5-2* in seedlings (Figure 2a and 2b), is causing co-suppression in adults. Therefore, although the *pex5-2* adult growth defects persist after backcrossing, we cannot definitively conclude that the *pex5-2* growth defects visible in 5-week-old plants are caused by the *pex5-2* mutation, and we have removed the adult plant photo from Figure 2c.

2. In Figure 4 (e), pex5-2 mutant showed that PEX5 protein level was not altered by the increasing temperature. The thiolase mature protein in the pex5-2 mutant is also similarly

expressed like wild-type. However, the PMDH (p) protein level is increased in the pex5-2 mutant and the authors did not point out this finding and what might have caused it.

Response: The reviewer is correct that we detect increasing PMDH (p) levels with increasing temperature in *pex5-2* (Figure 4e). We completely reworked the description of Figure 4 and clarified this section by adding explicit mention of this observation to the results as follows: "growth at 28°C resulted in slight accumulation of the precursor form of PMDH in light-grown *pex5-2* seedlings (Figure 4e), suggesting worsened PTS2 processing at high temperature, whereas PMDH processing was nearly complete in seedlings grown at 16°C (Figure 4e), suggesting improved PTS2 processing at low temperature."

3a. GFP-PTS1 was used to show the defect in PTS1 import. It would be interesting to check DEG15 protein level to provide more evidence to support PTS1 defective import in the pex5-2 mutant. Apart from DEG1, other protein like SOX and ICL also can be used to observe the defective PTS1 import.

Response: In an attempt to monitor DEG15 levels, we purchased a commercially available anti-DEG15 antibody and tested its specificity via immunoblotting. Unfortunately, in our hands this antibody does not detect any proteins in wild type that are absent in *deg15* null alleles. Our failure to validate this antibody means that we are unable to monitor DEG15 levels in our *pex5* mutants. GFP-PTS1 is considered a generic PTS1 protein because aside from the three-amino acid C-terminal PTS1 sequence, GFP-PTS1 does not have sequences from an endogenous peroxisomal protein that could lead to peroxisome entry via piggybacking, for example. Monitoring other PTS1 proteins could be interesting but would require immunofluorescence experiments or crossing additional reporter proteins into the mutants, which we feel are beyond the scope of the present work.

4. HSC70 did not work as good loading control because the band intensity was significantly varied in the same set of experiment. It would be better if the author could use some other proteins ACTIN, TUBULIN or some other reference proteins as a better loading control.

Response: We used HSC70 to monitor loading in the immunoblot experiments because the *HSC70/At5g02500* gene is expressed in many tissues at similar levels (AtGenExpress), the HSC70 protein is a different molecular weight than our experimental proteins, and the anti-HSC70 antibody was raised in mice instead of rabbits, which were the production host for most of our other antibodies. Although HSC70 levels do appear to vary a bit from lane to lane in some experiments, we believe that this minor variation reflects loading differences, which is what we are using this control antibody to monitor.

5. Although PEX5 was seen stabilized upon MG132 proteasome inhibitor treatment in previous reports it would be interesting to perform the in vitro ubiquitination assay to strengthen the claim of PEX5 ubiquitination and its subsequent degradation.

Response: We agree that direct detection of ubiquitinated PEX5 in vivo (as has been done in yeast, for example) or in vitro in the Arabidopsis system would be an interesting direction for future experiments. However, we do not report MG132 experiments in this manuscript and we suggest that examination of PEX5 ubiquitination is not needed to bolster the conclusions of the article and is thus beyond the scope of the present work.
